# A Comparative Study of Methyl-BEAMing and Droplet Digital PCR for *MGMT* Gene Promoter Hypermethylation Detection

**DOI:** 10.3390/diagnostics14222467

**Published:** 2024-11-05

**Authors:** Marco Macagno, Valeria Pessei, Noemi Congiusta, Luca Lazzari, Sara Erika Bellomo, Fariha Idrees, Alessandro Cavaliere, Filippo Pietrantonio, Alessandra Raimondi, Eleonora Gusmaroli, Maria Giulia Zampino, Lorenzo Gervaso, Davide Ciardiello, Giuseppe Mondello, Armando Santoro, Nicola Personeni, Emanuela Bonoldi, Maria Costanza Aquilano, Emanuele Valtorta, Salvatore Siena, Andrea Sartore-Bianchi, Alessio Amatu, Erica Francesca Bonazzina, Katia Bruna Bencardino, Guido Serini, Silvia Marsoni, Ludovic Barault, Federica Di Nicolantonio, Federica Maione

**Affiliations:** 1 Candiolo Cancer Institute, FPO-IRCCS, 10060 Candiolo, TO, Italy; marco.macagno@ircc.it (M.M.); valeria.pessei@hotmail.it (V.P.); noemi.congiusta@ircc.it (N.C.); saraerika.bellomo@ircc.it (S.E.B.); fariha.idrees@unito.it (F.I.); alessandro.cavaliere@unito.it (A.C.); guido.serini@ircc.it (G.S.); federica.dinicolantonio@ircc.it (F.D.N.); 2 Department of Oncology, University of Torino, 10043 Orbassano, TO, Italy; ludovic.barault@gmail.com; 3 IFOM ETS—The AIRC Institute of Molecolar Oncology, 20139 Milano, MI, Italy; luca.lazzari@ifom.eu (L.L.); silvia.marsoni@ifom.eu (S.M.); 4 Department of Medical Oncology, Fondazione IRCCS, Istituto Nazionale dei Tumori, 20133 Milan, MI, Italy; filippo.pietrantonio@istitutotumori.mi.it (F.P.); alessandra.raimondi@istitutotumori.mi.it (A.R.); eleonora.gusmaroli@istitutotumori.mi.it (E.G.); 5 Division of Gastrointestinal Medical Oncology and Neuroendocrine Tumors, European Institute of Oncology, IRCCS, 20139 Milano, MI, Italy; maria.zampino@ieo.it (M.G.Z.); lorenzo.gervaso@ieo.it (L.G.); davide.ciardiello@ieo.it (D.C.); 6 Medical Oncology and Hematology Unit, Humanitas Cancer Center, IRCCS Humanitas Research Hospital, 20089 Rozzano, MI, Italy; giuseppe.mondello@cancercenter.humanitas.it (G.M.); armando.santoro@cancercenter.humanitas.it (A.S.); 7 Medical Oncology Unit, ASST Garda, 25025 Manerbio, BS, Italy; nicola.personeni@asst-garda.it; 8 Department of Hematology, Oncology and Molecular Medicine, Grande Ospedale Metropolitano Niguarda, 20162 Milano, MI, Italy; emanuela.bonoldi@ospedaleniguarda.it (E.B.); mariacostanza.aquilano@ospedaleniguarda.it (M.C.A.); emanuele.valtorta@ospedaleniguarda.it (E.V.); 9 Niguarda Cancer Center, Grande Ospedale Metropolitano Niguarda, 20162 Milano, MI, Italy; salvatore.siena@unimi.it (S.S.); andrea.sartorebianchi@ospedaleniguarda.it (A.S.-B.); alessio.amatu@ospedaleniguarda.it (A.A.); ericafrancesca.bonazzina@ospedaleniguarda.it (E.F.B.); katia.bencardino@ospedaleniguarda.it (K.B.B.); 10 Department of Oncology and Hemato-Oncology, Università degli Studi di Milano, 20122 Milano, MI, Italy

**Keywords:** MGMT, DNAmethylation, Methyl-BEAMing, digital PCR, metastatic colorectal cancer

## Abstract

***Background: ****O-6-methylguanine-DNA methyltransferase* is responsible for the direct repair of O6-methylguanine lesions induced by alkylating agents, including temozolomide. *O-6-methylguanine-DNA methyltransferase* promoter hypermethylation is a well-established biomarker for temozolomide response in glioblastoma patients, also correlated with therapeutic response in colorectal cancer. ***Objectives:*** The ARETHUSA clinical trial aims to stratify colorectal cancer patients based on their mismatch repair status. Mismatch repair-deficient patients are eligible for treatment with immune checkpoint inhibitors (anti-PDL-1), whereas mismatch repair-proficient samples are screened for *O-6-methylguanine-DNA methyltransferase* promoter methylation to identify those suitable for temozolomide treatment. ***Methods:*** In this context, a subset of ARETHUSA metastatic colorectal cancer samples was used to compare two different techniques for assessing *O-6-methylguanine-DNA methyltransferase* hypermethylation: Methyl-BEAMing, a highly sensitive digital PCR approach that combines emulsion PCR and flow cytometry, and droplet digital PCR, a more automated procedure that enables the rapid, operator-independent analysis of a large number of samples. ***Results:*** Our study clearly demonstrates that the results obtained using Methyl-BEAMing and droplet digital PCR are comparable, with both techniques showing similar accuracy, sensitivity, and reproducibility. ***Conclusions:*** Digital droplet PCR proved to be an efficient method for detecting gene promoter methylation. However, the Methyl-BEAMing method has proved more sensitive for detecting low quantities of DNA.

## 1. Introduction

Temozolomide (TMZ) exerts its therapeutic effect by inducing DNA methylation at the N-7 and O6 positions of guanine and the N-3 position of adenine. O6-methylguanine (O6-meG) pairs with thymine instead of cytosine during DNA replication, thereby activating the post-replication mismatch repair (MMR) system, which leads to cell cycle arrest and apoptosis. The O6-methylguanine-DNA methyltransferase (MGMT) enzyme recognizes the O6-meG mismatch and repairs the lesion by transferring the alkyl group from guanine to a cysteine residue in its active site [1,2]. Epigenetic regulation through the methylation of the *MGMT* gene promoter prevents protein synthesis in cancer cells, consequently increasing their sensitivity to alkylating agents. Indeed, in glioblastoma patients, *MGMT* promoter hypermethylation is an established predictive biomarker for TMZ response and is approved for use in this cancer type [3,4]. Furthermore, *MGMT* promoter methylation is present in 30–40% of metastatic colorectal cancer (mCRC) cases and is directly correlated with increased DNA damage. Several phase 2 clinical studies have utilized *MGMT* methylation as a predictive and prognostic marker for TMZ response in chemotherapy-refractory mCRC [5]. Patients with high levels of promoter methylation exhibit longer disease-free survival upon TMZ treatment compared to those with reduced methylation levels and increased MGMT protein expression [6,7]. In general, the failure of the MMR system leads to the accumulation of DNA defects, and specifically in colorectal cancer (CRC), MMR dysfunction occurs in approximately 5% of total mCRC cases [7]. Based on MMR status, CRC patients are categorized as mismatch repair-proficient (MMRp) or mismatch repair-deficient (MMRd) [8,9]. A subset of MMRd CRC exhibits a microsatellite instability (MSI) phenotype, characterized by the accumulation of mutations in short, repetitive DNA regions known as microsatellites [10]. Patients with MSI-positive tumors have a better prognosis compared to those with microsatellite-stable (MSS) tumors and thus exhibit distinct histological features, which make them responsive to treatment with immune checkpoint inhibitors such as the anti-PD1 antibody pembrolizumab [11,12,13]. Based on previous studies, we contributed to a two-step clinical trial called ARETHUSA [14]. The trial begins with an initial screening in which MMRd mCRC patients are treated with pembrolizumab, while MMRp patients are assessed for MGMT protein expression and methylation status. Patients lacking MGMT protein and displaying promoter hypermethylation are selected for the first (priming) phase of the trial and treated with TMZ to induce a hypermutant status. Those patients who develop a tumor mutational burden (TMB) of ≥20 mutations per megabase (mut/Mb) upon progression after TMZ treatment proceed to the second phase, where they are treated with pembrolizumab as monotherapy [14]. Since *MGMT* promoter methylation status plays a critical role during the priming phase of this trial, it is essential to select and validate a sensitive and specific method for methylation analysis in order to minimize issues related to sampling and tumor heterogeneity. Traditionally, *MGMT* status has been assessed qualitatively using methylation-specific polymerase chain reaction (MSP) and bisulfite pyrosequencing. To improve the assessment of *MGMT* methylation, herein, we firstly employed a quantitative technique called Methylation Beads, Emulsion, Amplification, and Magnetic technology (Methyl-BEAMing) [15,16]. Methyl-BEAMing is considered one of the most reliable techniques for quantitatively evaluating *MGMT* promoter methylation [15]. In this study, we aimed to compare Methyl-BEAMing analysis with droplet digital PCR (ddPCR), a fully automated technology that may offer greater protection against operator-dependent errors, thus allowing the simultaneous analysis of many DNA samples in a shorter time period [17]. Our study demonstrates that both Methyl-BEAMing and ddPCR provide similar reproducibility, accuracy, and specificity for the quantitative assessment of *MGMT* methylation in clinical samples. However, Methyl-BEAMing showed higher sensitivity for detecting low quantities of DNA.

## 2. Material and Methods

### 2.1. DNA Extraction

FFPE tissues were sectioned using a microtome (Leica Biosystem, Nussloch, Germany) to obtain five 10 µm thick slices. Serial sections were cut, and IHC scoring was performed in a semi-quantitative fashion, taking into account both the extension and intensity of staining. Positive MGMT staining was defined as the staining intensity of the majority of tumor cells, as previously described [7]. To ensure an unbiased evaluation, at least two independent histologists scored the FFPE tissues and provided the cellularity values for each sample. Bulk DNA for molecular analysis was extracted from these tissues using the QIAamp DNA FFPE tissue kit (Qiagen, Hilden, Germany), following the manufacturer’s instructions. The protocol consists of six steps: the removal of paraffin, lysis using proteinase K, and heating at 90 °C to break the formalin-induced crosslinks. Subsequent steps included DNA binding, washing, and elution from the tissue.

### 2.2. DNA Quantification

DNA quantification was carried out using two methods: Nanodrop (DeNovix, Wilmington, DE, USA) and Qubit 4 (ThermoFisher, Waltham, MA, USA). Nanodrop quantifies nucleic acids via spectrophotometry by measuring the UV light absorption of nucleic acids; DNA concentration is directly proportional to the absorbed light. In contrast, Qubit is a highly sensitive fluorometer that selectively binds to nucleic acids, minimizing non-specific binding. For double-stranded DNA (dsDNA) quantification, we used two reagent types: the broad-range kit (2–1000 ng) and the high-sensitivity kit (0.2–100 ng) for dsDNA.

### 2.3. Treatment with Bisulfite

Bisulfite conversion was performed using the EZ DNA Methylation Gold kit (Zymo Research, Orange, CA, USA) according to the manufacturer’s protocol. Sodium bisulfite (HSO_3_^−^) from the CT Conversion Reagent was added to the DNA samples to distinguish between methylated and unmethylated cytosines. Upon incubation at high temperatures, unmethylated cytosines were converted to sulfonated cytosines and sulfonated uracils through deamination. In the final step, cytosines were irreversibly converted into uracils. DNA samples were then eluted from the columns using the elution buffer provided by the kit. Unmethylated cytosines were converted to uracils, whereas methylated cytosines remained unchanged due to the presence of a CH_3_ methyl group at the 5-carbon position.

### 2.4. Methyl-BEAMing Assay

The Methyl-BEAMing technique involves two rounds of DNA amplification: the first step uses specific primers for methylated and unmethylated DNA, while, in the second step, primers covalently bound to magnetic beads are compartmentalized into microdroplets. In Methyl-BEAMing, methylated beads are generated after the PCR amplification of individual DNA molecules in aqueous nanocompartments suspended in a continuous oil phase. The status of these beads is then detected by flow cytometry using fluorescent probes that specifically hybridize to methylated or unmethylated sequences. In contrast, ddPCR is a fully automated technique that uses nanodroplet sample partitioning, offering high sensitivity without the need for a standard curve. Bisulfite-converted DNA was analyzed using the Methyl-BEAMing technique to detect the methylation status of the MGMT gene promoter. The protocol begins with the initial polymerase chain reaction (PCR) amplification of the purified DNA using tagged primers to enrich the target locus. Amplicons were then diluted (1/16,000) and reamplified using tag-coated beads to enhance sensitivity. The second round of amplification was conducted in an emulsion, enabling the physical separation and independent amplification of different templates. PCR mixtures were prepared according to previously established conditions [15]. After amplification, the emulsion was broken using isopropanol/butanol, and the amplicons were hybridized with fluorescent probes specific to methylated or unmethylated bisulfite-converted templates. Flow cytometric analysis was performed using the Accuri C6 (BD Biosciences, Franklin Lakes, NJ, USA). The percentage of methylation was calculated by dividing the number of methylated events by the total number of specific events. A minimum of 200 cumulative events (methylated + unmethylated) were required to validate the results. All analyses were conducted in duplicate; final results were obtained by averaging the values, which were then normalized according to the percentage of tumor cells in the sample. A Positive Predictive Value of 0.67 and Negative Predictive Value of 0.98 had been determined previously with ROC analysis [15]. To check the quality of the assay, in each run, internal controls corresponding to 100%, 50%, and 0% methylation were used.

### 2.5. Droplet Digital PCR

Droplet digital PCR is a digital PCR method in which DNA samples are split into thousands of separate reaction droplets. PCR occurs in each droplet, which contains DNA molecules and fluorescent probes. A mixture of DNA, primers, and probes was prepared in a final volume of 20 µL. DNA samples and oil were loaded into the wells of the droplet generator. A vacuum system ensured that both the sample and oil passed through microfluidic circuits, forming a uniform dispersion of droplets. Each well containing 20 µL of mixture was divided into highly uniform compartments. The droplets were transferred to a 96-well PCR plate, and amplification was carried out in a conventional thermal cycler. After amplification, the plate was loaded into a reader where droplets passed through a two-color detector. Droplets were classified as positive or negative based on fluorescence amplitude, with the following binary threshold: 1 = positive, 0 = negative. For the ddPCR reaction, 5–10 µL of DNA template was mixed with 10 µL of ddPCR Supermix for Probes (Bio-Rad, Hercules, CA, USA) and 5 µL of the primer and probe mix (fluorophores FAM and HEX). The sample and mix were added to a cartridge along with 60 µL of oil required for droplet generation (Auto-DG, Bio-Rad). The droplets were transferred to a 96-well plate and subjected to temperature-dependent amplification in a thermal cycler. Finally, fluorescence was evaluated using the QX200 Droplet Reader (Bio-Rad).

### 2.6. Methylation Assay Controls

Ultramer oligomers of 250 bp (representing fully methylated or unmethylated bisulfite-converted templates) were used as positive controls. The specificity and sensitivity of ddPCR were verified using a synthetic methylation scale (ranging from 0% to 100% MGMT methylation) by mixing the two controls. Each amplification batch included two positive controls (methylated and unmethylated) and one negative control (no template).

### 2.7. Statistical Analyses

Statistical analyses, including correlation, linear regression, and Kappa statistics, were conducted using Prism 7.00 for Windows (GraphPad Software, La Jolla, MA, USA). Fisher’s exact test was performed to calculate the probability of the data for the 2 × 2.

## 3. Results

### 3.1. Methyl-BEAMing Analysis of MGMT Methylation

A subset of 342 samples from mCRC patients enrolled in the ARETHUSA trial was analyzed (Ethical Committee: Comitato Etico Milano Area 3 Italy; approval date 6 August 2018, IFOM-CPT002/2018/PO001; EudraCT number, 2018-001441-14). Formalin-fixed, paraffin-embedded (FFPE) tissue samples were previously evaluated for MGMT protein expression through immunohistochemistry (IHC). Samples with low-to-absent MGMT protein expression were processed to assess *MGMT* gene hypermethylation. This analysis was initially performed using Methyl-BEAMing, which involved two rounds of DNA amplification, thereby increasing the sensitivity of the protocol and enabling the detection of a small number of target molecules. Specific fluorescent probes for methylated or unmethylated DNA sequences were then used for flow cytometry analysis.

**Figure 1:**
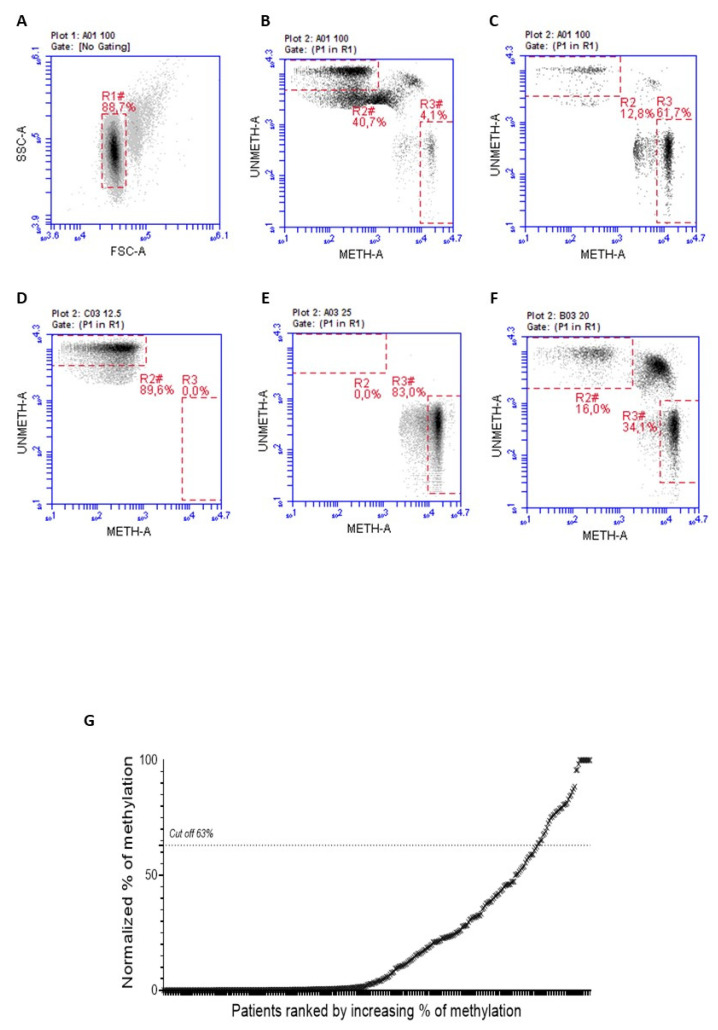
Representative plots of the gate strategy used for the flow cytometric analysis. (**A**) ARETHUSA samples analyzed through the Methyl-BEAMing technique were first grouped based on their complexity events. (**B**) Gate R2 (46.6%) includes unmethylated events, whereas R3 (4.1%) contains methylated ones. (**C**) The value of 61.7% referred to gate R3 is representative of a methylated sample. (**D**) Unmethylated sample control (R3 0%). (**E**) Methylated sample control (R3 83%). (**F**) The 50% control, obtained by mixing the unmethylated control with the methylated one (R3 34.1%) (**G**) Methylation profiling of CRC tissue samples assessed by Methyl-BEAMing.

MGMT-negative FFPE mCRC samples were first subjected to Methyl-BEAMing to determine the methylation levels of the *MGMT* gene promoter. A previous ROC analysis determined the cutoff value to distinguish between hypermethylated and unmethylated samples [7]. A threshold of 63% was set to identify positive samples and quantitatively predict the therapeutic response. After two rounds of PCR amplification, the promoter methylation status was detected by flow cytometry, using probes specific to methylated and unmethylated sequences. As shown in Figure 1A–F, gate R2 represents unmethylated events, while R3 contains methylated events; R2 and R3 values are normalized according to the percentage of cancer cells obtained from IHC analysis. The final graph from this analysis allowed us to classify samples as positive or negative based on the percentage of events in gates R2 and R3. Using the Methyl-BEAMing assay, we found that the *MGMT* promoter was methylated in 7.78% of cases (Figure 1G).

### 3.2. Droplet Digital PCR Analysis

**Figure 2:**
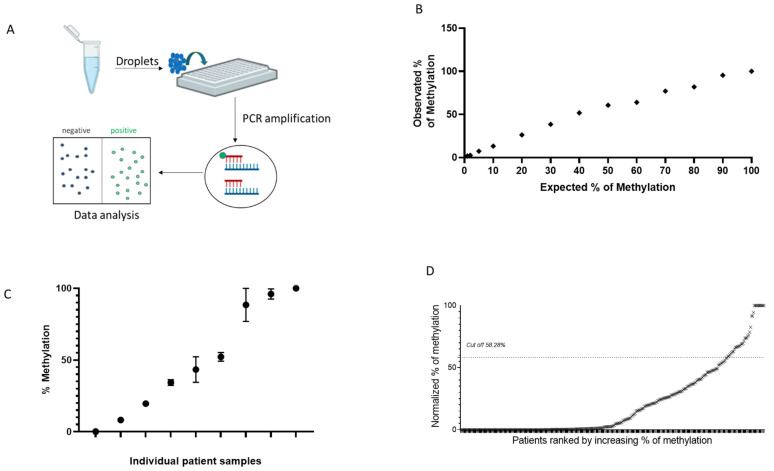
Assessment of *MGMT* gene promoter hypermethylation using the ddPCR technique. (**A**) Scheme of ddPCR: DNA amplification occurs independently in thousands of drops through a water-in-oil emulsion. Droplets are classified as positive or negative based on the emitted fluorescence wavelength. (**B**) Linearity of quantification of ultramer oligonucleotide mixture assessed by ddPCR. (**C**) Methylation analysis by ddPCR using three independent bisulfite treatments in nine different mCRC samples. (**D**) *MGMT* methylation profiling of CRC tissue samples obtained by ddPCR.

*MGMT* methylation analysis obtained by ddPCR is reported in Figure 2. Samples were processed through a two-color plate reader, where droplets were classified as positive or negative based on the emitted fluorescence (Figure 2A). ddPCR is one of the most precise and sensitive techniques, allowing the absolute quantification of DNA molecules in each sample. To assess the specificity and sensitivity of this method, a calibration curve was performed using an ultramer oligonucleotide mixture (Figure 2B). As shown by the linear curve, ddPCR analysis was highly reproducible and accurate. Reproducibility was further confirmed by performing three independent bisulfite treatments on separate mCRC samples (Figure 2C).

To evaluate the capability of ddPCR for *MGMT* methylation assessment, the same CRC samples previously analyzed using Methyl-BEAMing were tested with ddPCR. First, a new threshold value for distinguishing positive from negative samples was determined using the linear regression equation y = a × x + b, where the independent variable (x) corresponded to the cutoff used in the Methyl-BEAMing analysis. With the calculated cutoff value of 58.28%, we found that 10.28% of samples displayed *MGMT* gene methylation (Figure 2D). Notably, 17 samples were excluded from ddPCR analysis due to insufficient DNA, which could not be amplified, rendering them undetectable.

### 3.3. Methyl-BEAming and Droplet Digital PCR Comparison

**Figure 3:**
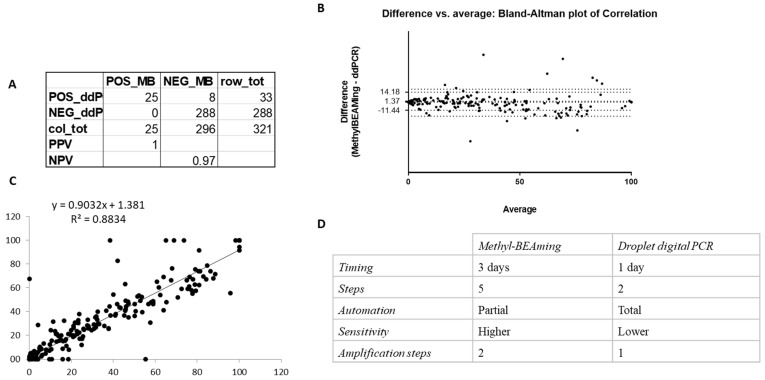
Evaluation of the concordance between results obtained using Methyl-BEAMing and those obtained using ddPCR. (**A**) Contingency table was carried out to qualitatively compare the number of methylated samples evaluated by either Methyl-BEAMing or ddPCR analysis. (**B**) Bland–Altman plot shows the agreement between the results of both techniques used. (**C**) Correlation and linear regression between Methyl-BEAMing and droplet digital PCR results. (**D**) The table reports differences between Methyl-BEAMing and ddPCR, including the time of execution, number of amplification steps, automation grade, and sensitivity.

To determine whether the number of hypermethylated samples detected by both Methyl-BEAMing and ddPCR was comparable (Figure 3), we created a contingency table and found that the specificity of both methods was very similar (Figure 3A). Notably, due to the inability of ddPCR to detect low DNA amounts, the comparison was conducted on only 321 samples. To assess the concordance between the two techniques for methylation detection, we constructed a Bland–Altman plot, which showed that the results obtained using Methyl-BEAMing and ddPCR were comparable, though Methyl-BEAMing was more sensitive for samples with a low DNA content (Figure 3B). Furthermore, linear regression analysis confirmed these findings, with a coefficient of determination (r^2^ = 0.8834, *p* < 0.0001), indicating that the independent variable (BEAMing data) could predict the dependent variable (ddPCR data) (Figure 3C). Despite the high concordance between the methods, differences between them were noted and are summarized in Figure 3D.

### 3.4. MGMT Methylation Is Increased in Aged Women with mCRC

**Figure 4:**
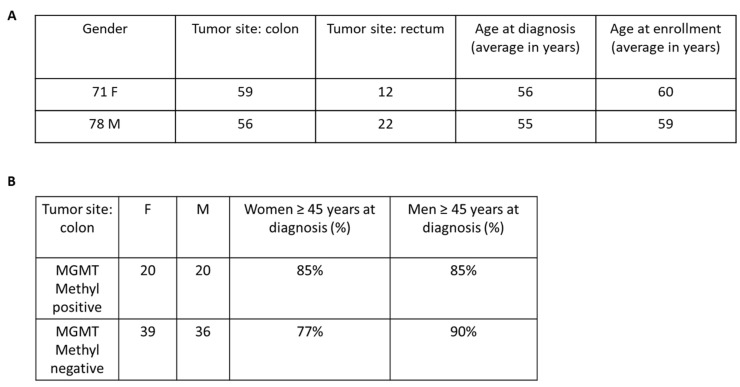
Analysis of the incidence of MGMT gene promoter methylation in a cohort of mCRC patients. (**A**) Clinical features collected for 148 mCRC patients. (**B**) This Table shows a non-statistically significant trend of increased *MGMT* methylation frequency in older women compared to younger ones (Fisher’s exact probability test one-tailed *p*-value = 0.4963).

To investigate whether *MGMT* methylation status had predictive value in tumor-bearing patients, we conducted a small-scale epidemiologic study using clinical data from the ARETHUSA trial (Figure 4). Unfortunately, for several patients, the Electronic Case Report Forms were not properly collected. Therefore, this analysis was performed on a smaller cohort of 148 patients for whom we had complete medical information (Figure 4). Based on previous studies suggesting a correlation between *MGMT* methylation and advanced age in colon cancer patients [18], we specifically analyzed this subgroup. Furthermore, we considered tumor location and subdivided the data into patients with either rectal or colon cancer. Although our analysis did not reach statistical significance, we observed an interesting trend showing that younger women had lower methylation frequencies compared to women over 45 years old (Figure 4B). Notably, this difference was not observed in male patients, further supporting a correlation between age, gender, tumor location, and increased *MGMT* methylation.

## 4. Discussion

Chemoresistance to standard therapy remains a significant challenge in the treatment of metastatic colorectal cancer (mCRC) patients. In this context, identifying new therapeutic targets and predictive biomarkers for pharmacological treatment remains an unmet clinical need. The hypermethylation of the *MGMT* gene promoter and the resulting inactivation of protein expression play a key role in the early stages of colorectal cancer (CRC) development, as it is associated with an increase in G>A point mutations in other cancer-associated genes, such as *KRAS* and *TP53* [4]. Moreover, *MGMT* promoter hypermethylation may increase sensitivity to alkylating agents like Dacarbazine and Temozolomide, as observed in patients with glioblastoma, advanced melanoma, and neuroendocrine tumors [6,19,20]. However, the efficacy of Temozolomide in mCRC remains controversial. Several phase II clinical trials enrolling MGMT-deficient mCRC patients did not show a clear improvement in progression-free survival or overall survival [21,22]. Notably, our data align with previous findings showing that older age and female gender are generally associated with higher levels of *MGMT* and/or *p16* gene methylation. Despite the analysis being conducted on a limited cohort of mCRC patients, the observed trend supports the idea that *MGMT* hypermethylation may play an important role in colorectal cancer. In recent years, clinical studies on glioblastoma and mCRC patients have used methylation-specific PCR (MSP) to assess *MGMT* gene methylation [23]. However, it has been demonstrated that while MSP-based selection is necessary, it is not sufficient for the optimal stratification of patients who may benefit from alkylating agent therapy. Thus, using MSP alone for *MGMT* molecular analysis may be inadequate and requires further investigation. Recent studies have applied novel digital methods for patient stratification, providing greater prognostic and predictive significance for TMZ treatment. Among these, Methyl-BEAMing technology has proven more effective in assessing *MGMT* promoter methylation in glioblastoma samples compared to MSP or pyrosequencing [19]. Similarly, a retrospective study on FFPE mCRC samples using both standard and digital methods demonstrated the efficacy of Methyl-BEAMing in predicting prognosis and therapeutic response. To date, the highest prediction accuracy (87%) for treatment response has been achieved by combining multiple techniques to assess *MGMT* gene status, including Methyl-BEAMing, mass spectrometry, and RNAseq [17]. Additionally, MGMT protein expression detection via immunohistochemistry (IHC) has been evaluated as an exploratory biomarker in several studies, which have shown a high concordance between MGMT protein expression and quantitative *MGMT* methylation assessed using Methyl-BEAMing. These findings suggest that combining different methods is crucial in achieving the most accurate prediction. Furthermore, MMR evaluation proved crucial in predicting the TMZ treatment response in mCRC patients. In the ARETHUSA study, MGMT protein expression and promoter hypermethylation were assessed in MSS/MMRp patients prior to TMZ treatment, which was used as a noncanonical strategy to convert immunologically “cold tumors” into “hot tumors”. In this regard, *MGMT* hypermethylation assessment was found to be a crucial factor in patient selection. Our study directly compares Methyl-BEAMing and droplet digital PCR (ddPCR) for the assessment of *MGMT* promoter methylation. Both methods share a similar mechanism of action: they are based on molecular compartmentalization to perform large-scale amplification and can detect very low amounts of the target sequence. The results were normalized to tumor cell counts (as estimated by a histopathologist), and statistical analyses were performed to assess assay concordance. The analysis demonstrated a good correlation between the two techniques, further confirmed by linear regression analysis and sample distribution on a Bland–Altman plot. The sensitivity and specificity values indicate that both methods effectively identify false-positive and false-negative samples. Specifically, ddPCR yields optimal results when sufficient initial DNA is present, while Methyl-BEAMing is better suited for evaluating *MGMT* methylation in samples with limited DNA. The two-phase amplification process in Methyl-BEAMing enhances protocol sensitivity, but it also increases the error rate due to the dilution of the amplification product. This issue can be mitigated by using the fully automated ddPCR technique.

Overall, our study clearly demonstrates that combining IHC with either Methyl-BEAMing or ddPCR offers an accurate stratification of mCRC patients, leading to better prognostic and predictive outcomes for Temozolomide treatment.

## Data Availability

The data that support the findings of this study are available upon request from the authors.
